# Biological and Cellular Functions of the Microdomain-Associated FWL/CNR Protein Family in Plants

**DOI:** 10.3390/plants9030377

**Published:** 2020-03-19

**Authors:** Sandra Thibivilliers, Andrew Farmer, Marc Libault

**Affiliations:** 1 Department of Agronomy and Horticulture, Center for Plant Science Innovation, University of Nebraska-Lincoln, Beadle Center, Lincoln, NE 68503, USA; sthibi@unl.edu; 2 National Center for Genome Resources, Santa Fe, NM 87505, USA; adf@ncgr.org

**Keywords:** FWL/CND, microdomains, plasma membrane, legume nodulation, plant development

## Abstract

Membrane microdomains/nanodomains are sub-compartments of the plasma membrane enriched in sphingolipids and characterized by their unique protein composition. They play important roles in regulating plant development and plant-microbe interactions including mutualistic symbiotic interactions. Several protein families are associated with the microdomain fraction of biological membranes such as flotillins, prohibitins, and remorins. More recently, GmFWL1, a FWL/CNR protein exclusively expressed in the soybean nodule, was functionally characterized as a new microdomain-associated protein. Interestingly, GmFWL1 is homologous to the tomato FW2-2 protein, a major regulator of tomato fruit development. In this review, we summarize the knowledge gained about the biological, cellular, and physiological functions of members of the FWL/CNR family across various plant species. The role of the FWL/CNR proteins is also discussed within the scope of their evolution and transcriptional regulation.

## 1. Role of Plasma Membrane Micro/Nanodomain in Plant Biology

Eukaryotic biological membranes consist of bilayers of lipids embedded with proteins. The lipid and protein compositions of biological membranes strongly influence their functions [1]. For instance, membrane micro/nanodomains (referred to in this review as microdomains) are sub-compartments of biological membranes well-characterized for their unique lipid and protein composition (i.e., microdomains are enriched in sterols and sphingolipids, and in specific classes of proteins such as stomatin, prohibitin, flotillin, and HflK/C proteins (also known as SPFH proteins)) [2,3,4]. Microdomains are not static lipid/protein islands in the plasma membrane; their dynamic reallocation in the plasma membrane plays a critical role in controlling cell morphology and physiology [5].

For instance, the polar accumulation of auxin in the root is under the control of auxin transporters including the PIN1, 2, and 3 proteins [6,7]. The polar localization of PIN1 and 3 is regulated by the sterol methyltransferase 1 protein [8], while the polar accumulation of the PIN2 transporter in the plasma membrane depends on sterol-enriched components [9,10]. Microdomains play a critical role in plant [11] and cell developments (e.g., polar elongation of specialized cells such as the tip growth of the root hair cells and pollen tubes [12,13,14]). They also control intercellular communications: a specific population of microdomains such as those enriched in the cell-signaling regulator tetraspanin are closely associated with plasmodesmata [15,16,17]. Microdomains control plant response to microbial inoculations including fungal and viral infections [18,19,20,21,22,23,24]. In the legume model *Medicago truncatula*, the subcellular localization of the microdomain-associated protein FLOTILLIN2 (FLOT2) depends on the recognition of the symbiotic bacteria *Sinorhizobium meliloti* [25,26]. More specifically, upon microbial inoculation or application of the bacterial signaling molecule named Nod factor, FLOT2 accumulates at the tip of the Medicago root hair cell. This accumulation plays a critical role in promoting the infection of the plant root hair cell by symbiotic bacteria [25,26]. Flotillin-like proteins also play a role in vesicle trafficking during arbuscular mycorrhization, a symbiosis between plants and mycorrhizal fungi [27]. In *M. truncatula*, the SYMREM1 remorin regulates the infection of the root by *S. meliloti* as well as nodule development [28]. Recently, a functional analysis revealed that the medicago FLOT4 and SYMREM1 proteins interact with the receptor LYK3 to promote root hair cell infection by *S. meliloti* [29]. In soybean, two remorins, GmREM1.1 and GmREM2.1, have distinctive roles during the nodulation process: GmREM1.1 is transiently expressed in nodule primordia while GmREM1.2 is specifically expressed in the nodule during its entire development [30]. RNAi-mediated silencing also revealed that GmREM1.2 controls nodule formation [30]. Similarly to *M. truncatula* FLOT2, another soybean protein, GmFWL1 (FW2.2-LIKE 1), is also translocated at the tip of the soybean root hair cells in response to the inoculation with *Bradyrhizobium diazoefficiens*, the soybean symbiotic nitrogen-fixing bacterium inoculation [31]. The punctuate subcellular localization of GmFWL1 in the plant plasma membrane and its interaction with many well-characterized microdomain-associated proteins including SPFH proteins support that GmFWL1 is associated with the microdomain fraction of the soybean plasma membrane [31].

## 2. Phylogeny of the *FWL/CNR* Gene Family in Plants

GmFWL1 belongs to the FWL/CNR gene family. Alpert et al. (1995) reported the biological function of the first member of the FWL/CNR family through a genetic analysis of the tomato fw2.2 (fruit weight 2.2) quantitative trait locus (QTL) [32]. This gene acts as a major negative regulator of tomato fruit size compared to other tomato fruit weight QTLs (i.e., up to 30% of the tomato fruit weight [33,34,35]). The FW2.2 gene encodes a small protein (22 kDa) enriched in cysteine residues and characterized by one transmembrane domain [31,36,37,38]. The functional characterization of the protein suggests that FW2.2 acts as a negative regulator of cell division supporting the repressive role of FW2.2 in controlling tomato fruit size [34,39]. A closer analysis of the temporal transcriptional activity of FWL2.2 suggests that the timing and the level of expression of FWL2.2 during fruit development tomato contributes to regulate tomato fruit size [40]. In addition to its temporal regulation, the spatial transcriptional activity of FW2.2 is restricted to the fruit epidermal and sub-epidermal cells. The division rate of these two cell types is critical in regulating tomato fruit size [41].

**Figure 1:**
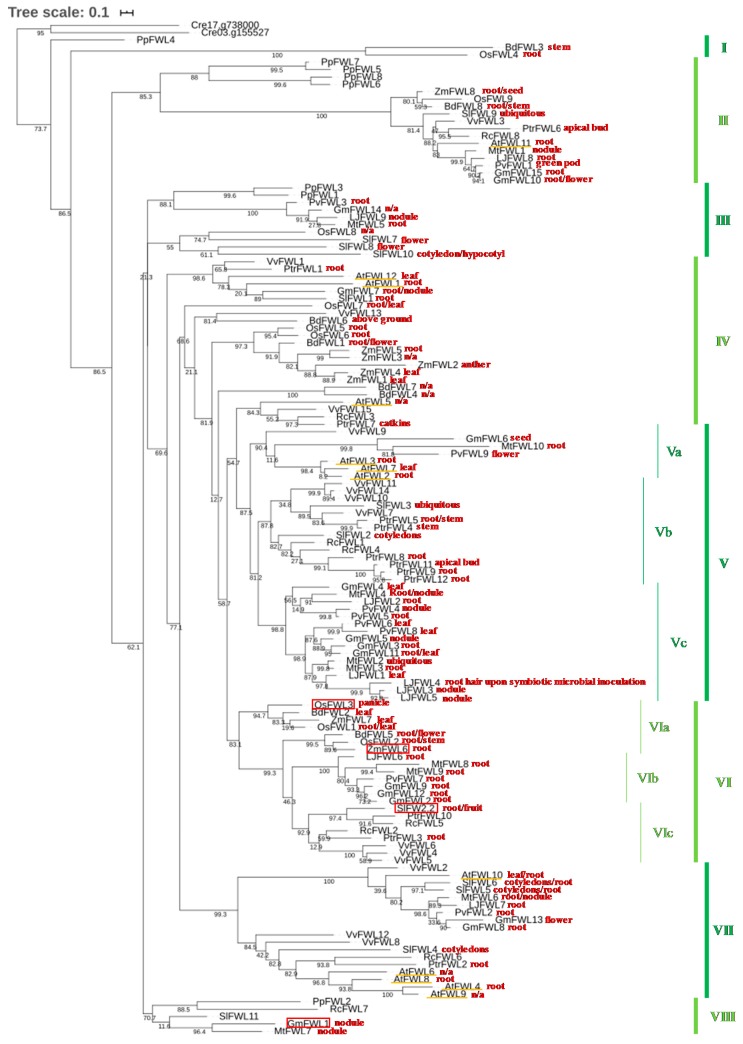
**Phylogenetic tree of the FWL/CNR family.** Members of the FWL/CNR family were identified with BlastP analysis against the genomic sequence of 13 different plant species. The 103 FWL/CNR protein sequences reported in Libault et al. (2010) [36] were used as queries in the Phytozome database (https://phytozome.jgi.doe.gov/; e-value ≤ 10e−20 and score ≥100). Alignment and phylogenetic reconstructions were performed using the function “build” of ETE3 v3.1.1 [61] as implemented on GenomeNet (https://www.genome.jp/tools/ete/). The multiple sequence alignment was performed using the CLUSTAL W Multiple Sequence Alignments Program with default parameters [62]. The tree was constructed using fasttree with slow NNI (Nearest Neighbor Interchange) and MLACC (maximum likelihood accuracy) = 3 (to make the maximum-likelihood NNIs more at nodes exhaustive) [63]. Values are SH-like local support. Two *Chlamydomonas reinhardtii* proteins (Cre17.g738000 and Cre03.g155527) were used to root the tree. The tree representation was generated using https://itol.embl.de/tree/. This tree was divided into eight major clades (I to VIII). Among them, Clades V and VI were divided into three distinctive subclades. Upon mining transcriptome atlases, we highlighted in red characters the tissue/organ where each FWL/CNR gene was the most expressed.

Taking advantage of the most recent releases of plant genomic sequences and their enhanced annotation [42], we looked for the FWL/CNR members across 13 different plant species (i.e., *Arabidopsis thaliana* [43], *Glycine max* [44], *Lotus japonicus* [45], *Medicago truncatula* [46], *Phaseolus vulgaris* [47], *Vitis vinifera* [48], *Solanum lycopersicum* [49], *Populus trichocarpa* [50], *Ricinus communis* [51], *Oryza sativa* [52,53], *Zea mays* [54], *Brachypodium distachyon* [55], and *Physcomitrella patens* [56]). These selected species cover different phyla of the plant kingdom including moss, monocotyledons, and dicotyledons. We identified 134 members in the FWL/CNR family (i.e., BlastP, e-value ≤ 10e−20 and score ≥100; Table S1). Among them, several members were characterized for their role in plant and fruit development across several mono- and di-cotyledons (Figure 1, red boxes). Notably, the maize gene cell number regulator1 (CNR1; ZmFWL6) negatively controls the overall plant size [57,58]. The biological function of other FWL/CNR genes as negative regulators of cell division and fruit development has also been described in *Oryza sativa* (OsFWL3; [38]), husk tomato (*Physalis floridana* CNR1; [37]), sweet cherry (*Prunus avium* CNR12 and CNR20; [59]), and in avocado (*Persea americana*; [60]).

To enhance our understanding of the evolution of the biological functions of the 134 members of the FWL/CNR family, we analyzed their phylogenetic relationships according to their amino acid sequences. These genes are distributed in eight major clades. Clade II is composed of FWL/CNR genes from all 13 species considered in this analysis, but the four *P. patens* FWL/CNR genes were clustered separately from the angiosperm genes. This result suggests that a FWL/CNR common ancestor existed before the speciation between angiosperms and mosses.

Interestingly, Clades V and VII are strictly composed of dicotyledon FWL/CNR genes. Upon dividing Clade V into three subclades (i.e., Subclades Va, b, and c), we noticed that Clades Vb and Vc were exclusively composed of non-legume and legume FWL/CNR genes (i.e., G. max, *M. truncatula*, *P. vulgaris*, and *L. japonicus*), respectively. Similarly, Clade VI can be divided into three different subclades: VIa, b, and c, which are exclusively composed of monocotyledonous genes, legume genes, and non-legume dicotyledonous FWL/CNR genes, respectively. These observations suggest that subsets of FWL/CNR genes evolved to likely gain unique biological functions in monocotyledons, and in legume and non-legume dicotyledons. Exploring the role of the genes in these different subclades (i.e., between Vb and c, and between VIa, b, and c) could provide interesting information regarding the evolution of their biological functions. OsFWL3, ZmCNR1 (ZmFWL6), and SlFW2.2, three genes of Clade VI, act as major regulators of plant and organ development (see above). GmFWL1, a regulator of soybean nodule development and bacterial infection, belongs to Clade VIII. Interestingly, MtFWL7 is evolutionary closely related to GmFWL1, suggesting that this *M. truncatula* gene might be the functional ortholog of GmFWL1. To date, there is no report of the function of MtFWL7 during *M. truncatula* nodulation; this may be a consequence of functional redundancies existing between members of the *M. truncatula* FWL/CNR gene family expressed in nodules (see below; Figure 1)

## 3. Transcriptional Regulation of the FWL/CNR Genes

The biological function of the GmFWL1 protein during soybean nodulation [36] depends on its interaction with many well-characterized microdomain-associated proteins including remorins, flotillins, and prohibitins [31]. Among the GmFWL1 protein partners, several are encoded by nodule or root-specific genes, suggesting a unique protein composition of the soybean nodule microdomain [31]. We assume that the differential transcriptional regulation of the genes encoding microdomain-associated proteins between cell, tissues, and organs influences the protein composition of microdomains and their biological activities. As a consequence, among other regulatory mechanisms, there is a need to better understand the transcriptional pattern of microdomain-associated genes and their co-expression.

Taking advantage of the release of various plant transcriptome atlases accessible via Phytozome, the Maize eFP Browser (http://bar.utoronto.ca/efp_maize/cgi-bin/efpWeb.cgi), the Lotus Base [64], the Tomato Functional Genomics Database (http://ted.bti.cornell.edu/cgi-bin/TFGD/digital/search.cgi?ID=D006), and targeted transcriptional studies (i.e., OsFWL genes [38]), we analyzed the transcriptional pattern of members of the FWL/CNR family. Overall, a large number of the FWL/CNR genes were mostly highly expressed in the root system of the plants. More rarely, these genes were significantly expressed in the canopy of the plants (Figure 1). Taking into consideration that microdomains are important regulators of the interactions between plant cells and microbes [23], the plant FWL/CNR genes preferentially expressed in the root system might play critical roles in controlling the interaction with soil microbes. Among them, a subset of the legume FWL/CNR genes, those preferentially expressed in the nodules (i.e., GmFWL1, 3, 5, and 7, MtFWL1, 4, 6, and 7, LjFWL3, 4, and 9, and PvFWL4), are likely specialized in regulating the interaction and infection of the legume root system by nitrogen-fixing rhizobia. Notably, the LjFWL4 gene is preferentially expressed in the root hair cells upon inoculation with symbiotic microorganisms. In soybean, GmFWL1 is also highly expressed in root hair cells during the early stages of the nodulation process [36]. However, contrary to LjFWL4, GmFWL1 is also expressed later during nodule development, suggesting that in *L. japonicus*, different FWL genes regulate the early and late stages of the nodulation process. Conversely, in soybean, GmFWL1 integrates both functions to promote the root hair cell infection by rhizobia and the establishment of the symbiosis between the plant nodule cells and the bacteria [31,36]. Looking at the phylogenetic distribution of these legume nodule-specific genes, they belong to six different clades (e.g., Clades II, III, IV, V, VII, and VIII). Hence, the nodule-specific legume FWL/CNR genes independently gained their unique transcriptional patterns and biological function.

In Clade VI, SlFW2.2, ZmFWL3/CNR, and OsFWL3 were functionally characterized by their role during fruit and seed development in tomato, maize, and rice, respectively [32,34,38,57,58], suggesting that other FWL/CNR members of this clade could also contribute to the fitness and development of other important crop species. By mining the transcriptome atlases, we noticed that many of the members of this clade were expressed in the plant root system (e.g., PvFWL7, GmFWL9, and 12, PtrFWL3, MtFWL8, and 9). Their putative role in root development should be further investigated.

**Figure 2:**
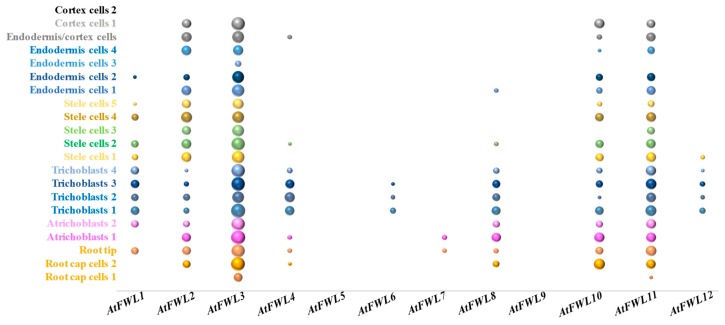
**Expression level of the 12 Arabidopsis thaliana FWL/CNR genes in various root cell types.** Each dot represents the log10-transformed expression levels of each AtFWL/CNR genes across 21 different cell cluster/cell type. Each cell cluster/cell type is represented by a different color. Root single-cell expression datasets were mined from Ryu et al., 2019 [65].

The protein composition of the microdomains could fluctuate between organs, tissues, and even cells based on the unique transcriptional activity of the genes encoding them (e.g., GmFWL1 protein partners are encoded by nodule and root-specific genes [31]). A more targeted analysis of the transcriptional activity of plant genes encoding microdomain-associated proteins would help to estimate the complexity and diversity of populations of microdomains in and between cell types, according to their protein composition. This statement is supported by microscopic studies conducted on plant plasma membranes [65]. The recent release of Arabidopsis root transcriptomes at a single-cell resolution now allows for a deeper exploration of gene expression and co-expression [66,67,68,69,70]. As a first attempt, we mined one of these Arabidopsis root single-cell transcriptomic datasets [68] to quantify the level of transcriptional activity of the twelve members of the AtFWL family. Consistent with previous bulk transcriptomic datasets showing that AtFWL2, 3, 10, and 11 are preferentially expressed in the root system, we found these four genes ubiquitously expressed across many root cell types (Figure 2), but with different levels of activity (i.e., AtFWL3 is more expressed than its three other homologs). AtFWL1, 4, and 8 expression is limited to a smaller number of root cell-types (Figure 2). For instance, AtFWL1 expression is strongly restricted to trichoblasts, atrichoblasts, and stele cells. Conversely, AtFWL5, 6, 7, 9, and 12 were found to be weakly expressed and only in a few cell types (Figure 2). AtFWL6 is exclusively expressed in trichoblasts (i.e., its transcript was detected in three out of the four sub-populations of trichoblasts; Figure 2) while AtFWL7 is expressed in one sub-population of atrichoblasts and in the root tip cells. The expressions of AtFWL5 and 9 were not detected in root cells, suggesting that these two genes do not have a function in Arabidopsis root microdomains. Similar analysis should be conducted for other families of genes encoding microdomain-associated proteins such as those encoding remorin, flotillin, and prohibitins. The integration of the transcriptional patterns of microdomain-associated genes at the single-cell level would help to estimate the complexity of microdomain population between cell-types.

## 4. Regulation of the Subcellular Localization of FWL/CNR Proteins

The sub-membrane localization of microdomain-associated proteins is influenced by external and internal stimuli. For example, during the nodulation process, MtFLOT2 and GmFWL1 are both characterized by their punctuate plasma membrane localization in the root hair cells of mock-inoculated plants. However, upon rhizobia inoculation, both proteins accumulate at the tip of the root hair cell [26,31]. This accumulation is critical to promote the infection of the root hair cells by the symbiotic bacteria. These observations clearly highlight the influence of microbes in reshaping membrane biology in order to promote plant cell infection by rhizobia.

Plant hormones also play an important role in controlling the translocation of microdomains in specific regions of the plasma membrane. Treatment of transgenic soybean roots clearly revealed that GmFWL1 and GmFLOT2 are both translocated at the tip of the root hair cells in response to auxin and salicylic acid [71], hormones regulating nodulation and plant-microbe interactions [72,73,74,75,76]. Conversely, cytokinin inhibits this re-location [71] and the infection of root hair cell by rhizobia [77,78,79,80]. Consequently, it is tempting to speculate that plant hormones control plant-microbe interactions [81,82,83,84,85,86] by controlling the distribution of microdomains in the plasma membrane.

## 5. Conclusions

Plant microdomains play critical roles in plant development and plant-microbe interactions. The emergence of the FWL/CNR gene family as a new family of microdomain-associated genes and their role in regulating plant development and plant-microbe interactions would justify a stronger focus on characterizing the biological and biochemical functions of these genes and proteins. Specifically, knowing that numerous crop FWL/CNR genes are preferentially expressed in the plant root system (Figure 1), one potential field of investigation would be the characterization of the function of FWL/CNR genes and the microdomains in controlling the interaction between the root cells and the root microbiomes. We noticed that a diverse population of AtFWL/CNR genes were highly and more specifically expressed in epidermal root cells (e.g., AtFWL4, 6, 8, and 12 are expressed in both trichoblasts and atrichoblasts; Figure 2). These genes could play important roles in controlling the interaction between plant and soil microbes, similarly to GmFWL1, which is a major regulator of soybean nodulation.

From a functional point of view, there is also a need to characterize the regulatory mechanisms controlling the activity of the FWL/CND proteins. Post-translational modifications of microdomain-associated proteins control their subcellular localization and biological function. For instance, the S-acylation of the rice remorin RbREM1 [87], the Arabidopsis receptor like-kinase FLS2 [88], and the N-glycosylation of the RIM21 yeast protein [89] interfere with their localization in the microdomains. In more detail, after binding to its ligand, the Arabidopsis FLS2 protein is deacylated, leading to its re-localization and autophosphorylation. This active form of the FLS2 protein triggers downstream signaling to promote the plant innate immune response [88]. Hence, we assume that the reversibility of some post-translational modifications could also act as a major regulator of the localization, conformation, mobility, and function of the FWL/CNR proteins, allowing a rapid adaptive response to environmental stresses. For instance, post-translational modifications of the GmFWL1 protein could affect its interaction with other microdomain-associated proteins such as prohibitins. Hence, there is a clear need to better characterize the biochemical properties of the FWL/CND proteins to fully understand their role in plant cell biology.

## Supplementary Materials

The following are available online at https://www.mdpi.com/2223-7747/9/3/377/s1, Table S1: Annotation and amino acid sequences of 134 FWL/CNR proteins identified across 13 different plant species.
